# Practical considerations on redesigning internal quality control process to verify the traceability of clinical results for decision-making

**DOI:** 10.5937/jomb0-45468

**Published:** 2024-06-15

**Authors:** Weizhi Cheng, Zhiwei Zhou, Shengkai Yan, Ye Jiang, Weichong Yang, Kunxue Shen, Richard Pang

**Affiliations:** 1 Qualab Biotech. Co., Ltd., Shanghai, P.R. China; 2 Zunyi Medical University, The Affiliated Hospital, Department of Laboratory Medicine, Zunyi, P.R. China; 3 Zunyi Medical University, School of Laboratory Medicine, Zunyi, P.R. China; 4 ProQ Consulting Services, Hong Kong, P.R. China

**Keywords:** quality control, laboratory management, analytical systems, traceability, kontrola kvaliteta, laboratorijski menadžment, analitički sistemi, sledljivost

## Abstract

Traceability is an important tool in the harmonization and standardization of reporting laboratory results, making them comparable across measurement systems. Driven by International Standardization Organization (ISO) 15189 accreditation requirements, medical laboratories have entered the era of metrological traceability. Although calibrators are a key component in the entire metrological traceability system, there is controversy over internal quality control (IQC) materials. It has been proposed that trueness materials supplied by the system's manufacturer with metrological traceability can be used to confirm that the performance of the measuring system is properly unbiased. This article focuses on the implementation challenges and operational hurdles of applying traceability concepts to IQC materials for trueness verification in medical laboratories regarding the most recent 2022 edition of ISO 15189 standard requirements for IQC and metrological traceability. There are practical considerations concerning the acquiring of IQC materials. We must acknowledge the limitations and restrictions that manufacturers and laboratories face before the recommendations can be applied in routine practices.

## Abstract (sr)

Sljedivost je važan alat za harmonizaciju i standardizaciju izvještaja o laboratorijskim rezultatima, čineći ih uporedivim u svim sistemima mjerenja. Vođene zahtevima za akreditaciju Međunarodne organizacije za standardizaciju (ISO) 15189, medicinske laboratorije su ušle u eru metrološke sledljivosti. Iako su kalibratori ključna komponenta u celom sistemu metrološke sledljivosti, postoje kontroverze oko materijala za internu kontrolu kvaliteta (IKK). Predloženo je da se materijali za istinitost koje je isporučio proizvođač sistema sa metrološkom sledljivošću mogu koristiti za potvrdu da su performanse mernog sistema ispravno nepristrasne. Ovaj članak se fokusira na izazove implementacije i operativne prepreke primene koncepata sledljivosti na IKK materijale za verifikaciju istinitosti u medicinskim laboratorijama u vezi sa najnovijim zahtevima standarda ISO 15189 za IKK i metrološku sledljivost iz 2022. godine. Postoje praktična razmatranja u vezi sa nabavkom IKK materijala. Moramo da priznamo ograničenja sa kojima se suočavaju proizvođači i laboratorije pre nego što se preporuke mogu primeniti u rutinskoj praksi.

## Introduction

Traceability is an important tool in the harmonization and standardization of reporting laboratory results, making them comparable across measurement systems. Driven by the International Standardization Organization (ISO) 15189 accreditation, medical laboratories have entered the era of traceability. ISO standards are being used to standardize practices globally [1]. The reliability of the measuring system is essential to decision-making, including accuracy and precision. Internal quality control (IQC) involves analyzing samples to find out if they meet the criteria for acceptability. For medical laboratories, although calibrators are a key factor in the entire quantitative traceability system [2], there is controversy over IQC materials. Recently, a recommendation has been proposed for redesigning the structure of IQC in the era of traceability [3]. However, the challenge for the medical laboratory is finding a suitable trueness control material to develop a specific strategy that permits verifying the traceability of clinical results in realtime and, most importantly, how to incorporate it into its IQC plan. The discussion will focus on the implementation challenges and operational hurdles of applying traceability concepts to IQC materials for trueness verification in medical laboratories, regarding the most recent 2022 edition of ISO 15189 standard requirements for IQC and metrological traceability.

## Implementation challenges

### Do quality control materials need to have metrological traceability?

Medical laboratories usually use two types of quality controls: IQC and inter-laboratory quality assessment (PT/EQA). The purpose of IQC is to monitor the testing systems imprecision, while PT/EQA is used to evaluate its accuracy [4]. It is worth noting that the ISO 15189:2022 accreditation document does not require the metrological traceability of IQC materials [1]. Independent or third-party QC materials are an important component of IQC programs. They are used to monitor the reliability of the measuring system and to estimate the measurement uncertainty of provided results. Medical laboratories treat IQC materials as patient samples during testing, and the traceability of their measurements is the same as that of patient samples. When IQC materials are tested on a specific analytical system, such as Abbott Alinity, the resulting measured value is traced back to that system. When testing the same IQC materials on Roche Cobas, the measured value can be traced back to it. However, if the IQC materials are tested on an analytical system with no traceability guarantee, their measured values are naturally not traceable. When it comes to independent third-party controls that are commonly used in laboratories, such as Bio-Rad, Randox, Technopath, and/or Thermo Fisher, the situation can be more complex. The assignment value is obtained using multiple analytical systems and/or different reagent systems. As a result, different analytical systems have corresponding assignment information on the assignment list or package inserts of such controls (Table 1). According to clause 6.5.3 »Metrological traceability of measurement results« of ISO 15189:2022 i.e., »*the laboratory shall establish and maintain metrological traceability of its measurement results by means of a documented unbroken chain of calibrations, each contributing to the measurement uncertainty, linking them to an appropriate reference*« [1]. So, information on traceability to a higher-order reference material or calibration procedure should be provided by an examination system manufacturer but not the IQC material provider.

**Table 1:** Measurement traceability information of different Chemistry Analyzer Systems^*1^ ^*1^ Taken from reagent package inserts of individual instrument manufacturers.<br>^*2^ Reference methods and reference materials are adopted in the database of the Joint Committee for Traceability in Laboratory Medicine (JCTLM).<br>https://jctlm.org/about-us/ (Accessed 9 October 2023)

Chemistry Analyzer<br>Systems	In-kit Control<br>Materials	Measurement Traceability<br>(Reference Methods and or Reference Materials^*2^)		
		Cholesterol	HbA1c	Creatinine
Abbott ARCHITECT	No	CDC Abell-Kendall Method	IFCC Method	ID/MS
Abbott Alinity	No	CDC Abell-Kendall Method	IFCC Method	ID/MS
Beckman Coulter AU	Yes	CDC Abell-Kendall Method	IFCC Method	ID/MSNISTSRM967
Ortho VITROS	Yes	CDC Abell-Kendall MethodNIST<br>SRM 911	IFCC Method	ID/MSNIST SRM914
Roche Cobas	Yes	CDC Abell-Kendall<br>MethodID/GC/MS	IFCC Method	ID/MS
Siemens Atellica	No	CDC Abell-Kendall Method	IFCC Method	ID/MS

Medical laboratories can participate in inter-laboratory programs that meet specific metrological criteria. ISO/IEC 17043:2023 requires organizers of inter-laboratory PT/EQA to consider the traceability and measurement uncertainty of their PT/EQA programs as far as possible [5]. The primary objective of an External Quality Assessment (EQA) program is not to evaluate the bias of the testing system. The measurement of bias necessitates a comprehensive analysis and statistical calculations. It is important to note that some PT/EQA programs may not have traceable reference values for their survey samples. However, according to clause 7.3.7.3 requirement of acceptable alternatives EQA program in ISO 15189:2022 i.e., *»when an EQA program is either unavailable or not considered suitable, the laboratory shall use alternative methodologies to monitor examination and method performance. The laboratory shall justify the rationale for the chosen alternative and provide evidence of its effectiveness. NOTE Acceptable alternatives include:*

*– analysis of a different lot number of the manufacturer'send-user calibrator or the manufacturer'strueness control material«* [1].

Since IQC materials require more frequent testing than PT/EQA QC materials, it is generally not feasible to use a single QC material to meet all needs. In this context, the following discussion will focus on the feasibility of using IQC materials in the era of traceability.

### Why is the commutability of QC materials so important?

Commutability refers to the ability of a material to behave in the same way as a patient sample when tested by different methods or laboratories. When commutable samples are used in PT/EQA programs, they can help identify differences between laboratories using the same or different methods for the same measurand. This is important because differences may arise due to the traceability of the calibrators, assay imprecision, differences in analytical specificity, and individual laboratory practices. In addition, the commutability of reference materials, including PT/EQA material, is important because it allows laboratories to monitor the traceability of manufacturers’ methods and the ability of laboratories to share results or reference intervals. It's important to note that the PT/EQA material needs to be suitable for effectively identifying these issues at a reasonable cost. One of the challenges for PT/EQA providers is to develop or obtain appropriate material. Ideally, the material should be commutable, with target values assigned from reference methods that use certified reference materials (CRMs).

### Are the quantity values for IQC materials metrologically traceable?

To ensure traceability, in vitro diagnostics (IVD) device manufacturers should define a calibration hierarchy to assign traceable values to their system calibrators. Medical laboratories should know and verify how manufacturers have implemented the traceability of their calibrators and estimate the corresponding measurement uncertainty on clinical samples [5]. End-user calibrators and in-kit QC materials often have similarities, but end-user calibrators have certified values assigned to them, while in-kit QC materials have nominal or target values. However, it may not be good practice to rely solely on the target values assigned to in-kit QC materials for verifying accuracy. This is because end-user calibrators may lack commutability, and manufacturers often do not provide sufficient information about calibration chains. Consequently, we may have to rely on lower-level measurement procedures without higher-order reference methods, which undermines the goal of maintaining an uninterrupted traceability chain that demonstrates the accumulation of uncertainty. The total uncertainty must remain acceptable to preserve clinical relevance since results cannot be transferred within one institution and method or between institutions and methods without it.

## Operational hurdles

### Does the traceability of the quantity of IQC materials have practical value for the laboratory?

**Figure 1:**
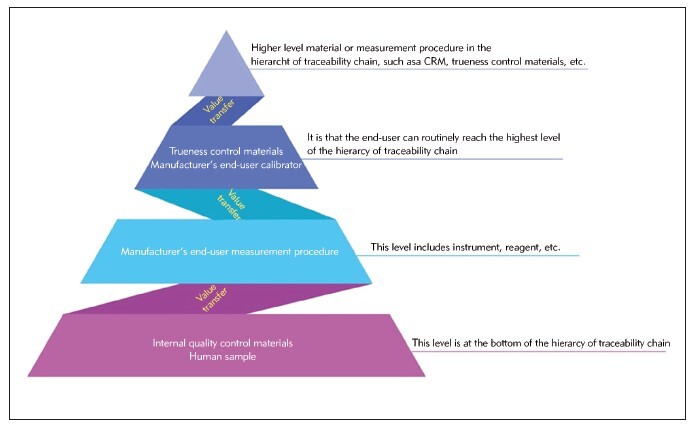
Hierarchy of traceability chain.

To put it simply, can we use the IQC materials' target value to evaluate the accuracy of the measurement process? To comprehend the problem better, it is crucial to recognize the role and importance of IQC materials in the traceability chain, as shown in Figure 1. When the value obtained from a medical laboratory test is compared to that of an IQC material, the IQC material is considered to be at the lowest level of the measurement traceability hierarchy. This is because its value corresponds to the test result of the patient sample. Therefore, IQC cannot be used as the basis for verifying the accuracy of the measurement. Instead, daily IQC results should be used only for monitoring precision.

### The situation is relatively complicated for the expected assignment of QC materials by QC material manufacturers.

Manufacturers of IVD equipment are obligated to ensure that their analytical systems are metrologically traceable to higher-order references. As per ISO 17511:2020, manufacturers can produce a »working calibrator« that can transfer calibration accuracy to end-user calibrators. This working calibrator must have a value that can be traced back to a reference measurement procedure (RMP) or a primary reference material (PRM). Once the initial calibrator is created, it can be used to calibrate other calibrators that are employed to calibrate the system. Manufacturers can even use a different lot number of the end-user calibrator to create the working calibrator [6]. To establish metrological traceability values assigned to the provided calibrators and in-kit QC materials for a specific instrument, manufacturers need to meet certain requirements. When assigning a specific value to the control, the crucial factor to consider is whether the manufacturer's assignment value transfer procedure satisfies the requirements for assigning target values to the QC materials. The IVD industry relies on reference materials, measurement procedures, and calibration laboratories to provide traceable calibrators and controls for routine laboratories. According to the authors’ understanding, when manufacturers assign the value of a QC material, they are usually only used as an IQC tool i.e., for checking the imprecision. It is important to consider the position of the metrological traceability level of IQC materials and patient samples on the calibration hierarchy. This level is the lowest, as shown in Figure 1. Therefore, even if target values have been assigned to the IQC materials, they cannot be used as a basis for verifying trueness.

## Discussion

Important concepts of metrological traceability and the estimation of measurement uncertainty are required for medical laboratories accredited by ISO 15189. Traceability is an important tool in the harmonization and standardization of reporting laboratory results [7]. The laboratory holds the responsibility to ensure and confirm the metrological traceability of the measuring systems they used, making their patient results comparable across measurement systems. The medical laboratory's challenge is finding a suitable trueness control material and how to incorporate it into its IQC plan. The ideal approach is to employ currently available CRMs recommended by international, national, and professional organizations, for the calibration of individual assays, and then the patient results are comparable to a reference through documented metrological traceability. The application of CRMs for PT/EQA purposes is well recognized. PT/EQA programs aiming for assessing accuracy organized by professional bodies or IVD industries could offer commutable samples with target values assigned by respective reference methods. Unfortunately, given the global economic situation, the current practice of metrological traceability applications in many PT/EQA programs is also not adequate. CRMs and IQC trueness control materials and the critical levels or concentrations are not available for the clinical applications of analytes measured in medical laboratories, irrespective of the global geographical regions. For certain sample matrix types, such as urine and other body fluids, the analytes needed might not be available or certified.

## Conclusions

There are practical challenges to consider when obtaining materials for IQC. There may be a lack of appropriate reference materials for certain matrix types used in diagnostic laboratory medicine and regular IQC testing of a larger number of analytical test runs may be too costly using these materials. Additionally, some materials may not be stable for a longer time for the measurand of interest, particularly for independent third-party QC materials with multiple constituents, the values given for each analyte of the in-kit control must be stable for use until the expiration date, on the condition of storage at the maximum allowable temperature for the stated period, Implementing IQC materials for trueness verification in medical laboratories can be challenging in terms of operation. Furthermore, the limited availability and high cost of trueness control materials make it impractical for many laboratories. Therefore, it is crucial to acknowledge the constraints that manufacturers and laboratories face before incorporating these recommendations into routine practices. It is essential to balance the benefits and limitations of redesigning IQC materials to verify the traceability of clinical results for decision-making.

## Dodatak

### Conflict of interest statement

All the authors declare that they have no conflict of interest in this work.
